# Swimming exercise increases serum irisin level and reduces body fat mass in high-fat-diet fed Wistar rats

**DOI:** 10.1186/s12944-016-0263-y

**Published:** 2016-05-13

**Authors:** Yun Lu, Hongwei Li, Shi-Wei Shen, Zhen-Hai Shen, Ming Xu, Cheng-Jian Yang, Feng Li, Yin-Bo Feng, Jing-Ting Yun, Ling Wang, Hua-Jin Qi

**Affiliations:** Jiangsu Provincial Taihu Lake Rehabilitation Hospital of Jiangsu Provincial People’s Hospital Group, Wuxi City, Jiangsu Province 214086 China; Wuxi No. 2 People’s Hospital Affiliated to Nanjing Medical University, Wuxi City, Jiangsu Province 214002 China; Jiangsu Institute of Parasitic Diseases, Wuxi City, Jiangsu Province 214064 China; Jiangsu Provincial Taihu Cadre’s Sanatorium of Jiangsu Provincial People’s Hospital Group, Wuxi City, Jiangsu Province 214086 China

**Keywords:** Swimming exercise, Irisin, Dual-energy X-ray absorptiometry (DXA), Visceral fat

## Abstract

**Background:**

It has been shown that irisin levels are reduced in skeletal muscle and plasma of obese rats; however, the effect of exercise training on irisin level remains controversial. We aim to evaluate the association of swimming exercise with serum irisin level and other obesity-associated parameters.

**Methods:**

Forty healthy male Wistar rats were randomly assigned to 4 groups: a normal diet and sedentary group (ND group), normal diet and exercise group (NDE group), high-fat diet and sedentary group (HFD group), and high-fat diet and exercise group (HFDE group. After 8 consecutive weeks of swimming exercise, fat mass and serum irisin level was determined.

**Results:**

Higher serum irisin levels were detected in the HFDE group (1.15 ± 0.28 μg/L) and NDE group (1.76 ± 0.17 μg/L) than in the HFD group (0.84 ± 0.23 μg/L) or the ND group (1.24 ± 0.29 μg/L), respectively (HFDE group vs. HFD group, *P* < 0.05; NDE group vs. ND group, *P* < 0.01). Pearson’s correlation analysis showed that serum irisin level negatively correlated with TG level (*r* = −0.771, *P* < 0.05), percentage fat mass (*r* = −0.68, *P* < 0.05), fat mass (*r* = −0.576, *P* < 0.05), visceral fat mass (*r* = −0.439, *P* < 0.05) and TC level (*r* = −0.389, *P* < 0.05). The fat mass, visceral fat mass and percentage fat mass were lower in the HFDE group than the HFD group (all *P* values < 0.01).

**Conclusion:**

Swimming exercise decreases body fat mass in high-fat-fed Wistar rats, which may be attributable to elevated irisin levels induced by swimming exercise.

## Background

Obesity is recognized as a worldwide health concern. Regular physical activity is found to play a key role in reducing the risk of obesity by increasing energy expenditure, although the detailed mechanism of which remains unclear. Skeletal muscle is the major target organ that participates in exercise. It is widely accepted that a motility factor is released during skeletal muscle contraction, which may improve muscle function. Irisin, a novel hormone secreted by muscle identified in 2012, is a peroxisome proliferator-activated receptor gamma coactivator 1 alpha (PGC-1α)-dependent myokine, and PGC-1α has been found to mediate uncoupling protein-1 (UCP-1) expression and brown fat thermogenesis, and to control mitochondrial biogenesis and oxidative metabolism in multiple cell types [1]. Importantly, irisin induces browning of subcutaneous white adipocytes and UCP-1-mediated thermogenesis. It has been shown that irisin levels are reduced in skeletal muscle and plasma of obese rats; however, the effect of exercise training on irisin level remains controversial. Plasma irisin concentration has been reported to decrease by 72 % in PGC-1α-deficient mice, whereas the concentration was significantly elevated (65 %) after the mice were subjected to 3 weeks of free wheel running [2]. In addition, it has been found that exercise training does not affect skeletal muscle fibronectin type III domain containing 5 (FNDC-5) or plasma irisin in obese rats [3]. The major purpose of this study was to evaluate the association of swimming exercise with serum irisin level and other obesity-associated parameters in high-fat-diet fed Wistar rats.

## Results

### Changes in body weight, body size, abdominal circumference, Lee’s index, blood lipid and glucose concentrations and serum insulin level

**Fig. 1:**
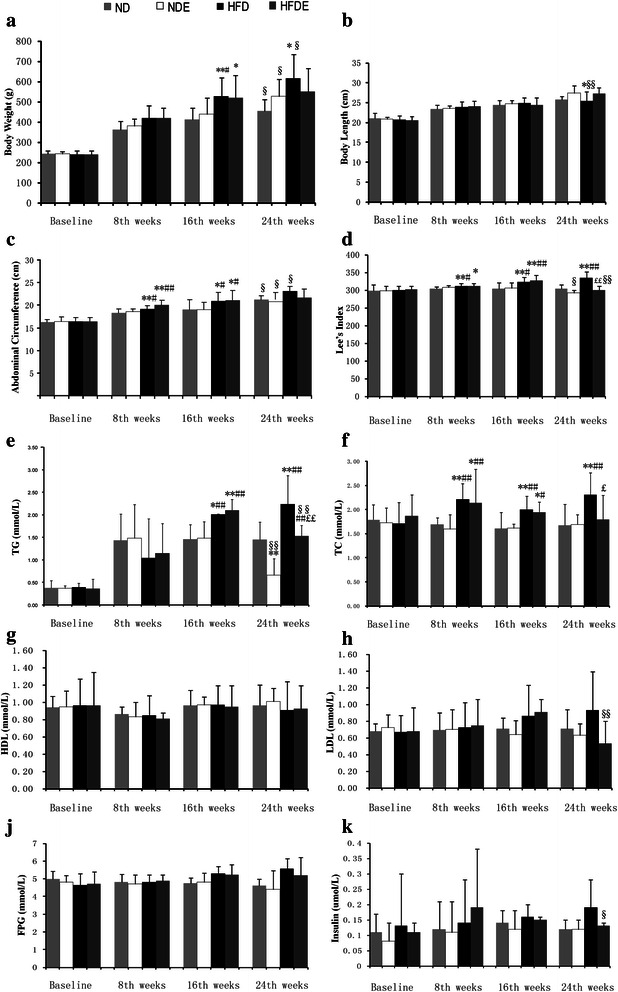
The body weight, Body length, Abdominal circumference, Lee’s index, Blood lipid, FPG and insulin values in all 4 groups. Body weight, abdominal circumference, Lee’s index and TC level were higher in the HFD (high-fat diet and sedentary group) and HFDE (high-fat diet and exercise group) groups than in the ND (normal diet and sedentary group) and NDE (normal diet and exercise group) groups (all *P* values < 0.05) after 8 weeks of diet feeding, and higher body weight, abdominal circumference, Lee’s index, TC and TG levels were al recorded in the HFD and HFDE groups compared to the ND and NDE groups at 16 weeks (all *P* values < 0.05). At 24 weeks (after swimming exercise), Lee’s index, TG and TC levels were lower in the HFDE group than in the HFD group (all *P* values < 0.05). In addition, body weight, abdominal circumference, Lee’s index, TG and TC levels were higher in the HFD group than in the ND and NDE groups (all *P* values < 0.05). Significantly reduced Lee’s index, serum TG, LDL and insulin levels (all *P* values < 0.05) and significantly increased body size (*P* < 0.05) in the HFDE group after swimming exercise in relation to the values before swimming exercise, while significantly decreased Lee’s index and TG level (all *P* values < 0.05) and significantly increased body weight, body size and abdominal circumference (all *P* values < 0.05) were found in the NDE group after swimming exercise as compared to those before swimming exercise (Fig. 1). *, *p* < 0.05 compared with ND and **,*p* < 0.01 compared with ND;#, *p* < 0.05 compared with NDE and ##, *p* < 0.01 compared with NDE;£,*p* < 0.05 compared with HFD and ££,*p* < 0.01 compared with HFD;§, *p* < 0.05 compared with 16th, §§, *p* < 0.01compared with 16th

There were no significant differences in body weight, body size, abdominal circumference, Lee’s index, blood lipid and glucose concentrations and serum insulin level at baseline among the four groups (*P* > 0.05). Body weight, abdominal circumference, Lee’s index and total cholesterol (TC) level were higher in the high-fat diet and sedentary group (HFD) and high-fat diet and exercise group (HFDE) than in the normal diet and sedentary group (ND) and normal diet and exercise group (NDE) (all *P* values < 0.05) after 8 weeks of diet feeding, and higher body weight, abdominal circumference, Lee’s index, TC and triglyceride (TG) levels were all recorded in the HFD and HFDE groups compared to the ND and NDE groups at 16 weeks (all *P* values < 0.05). At 24 weeks (after swimming exercise), Lee’s index, TG and TC levels were lower in the HFDE group than in the HFD group (all *P* values < 0.05); the body weight, abdominal circumference, LDL concentration, fasting blood glucose (FBG) and serum insulin level exhibited lower in the HFDE group than those in the HFD group, although no statistically significant differences were observed in body weight among the three groups (all *P* values > 0.05). In addition, body weight, abdominal circumference (AC), Lee’s index, TG and TC levels were higher in the HFD group than in the ND and NDE groups (all *P* values < 0.05). Paired *t*-tests revealed significantly reduced Lee’s index, serum TG, LDL and insulin levels (all *P* values < 0.05) and significantly increased body size (*P* < 0.05) in the HFDE group after swimming exercise in relation to the values before swimming exercise, while significantly decreased Lee’s index and TG level (all *P* values < 0.05) and significantly increased body weight, body size and abdominal circumference (all *P* values < 0.05) were found in the NDE group after swimming exercise as compared to those before swimming exercise (Fig. 1).

### Comparison of serum irisin level and fat parameters at 24 weeks

The serum irisin level exhibited significantly higher in the NDE group than the ND, HFDE and HFD groups (all *P* values < 0.05), while which was greater in the ND and HFDE groups than in the HFD group (both *P* values < 0.05).

**Fig. 2:**
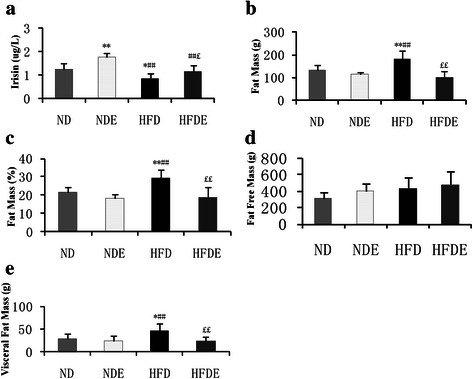
Comparison of serum irisin level and fat parameters among groups at 24 weeks. Significant differences in serum irisin level, fat mass, percentage of fat mass and visceral fat mass among the four groups (all *P* values < 0.05). A significantly higher serum irisin level was detected in the NDE (normal diet and exercise group) group compared to the ND (normal diet and sedentary group), HFDE (high-fat diet and exercise group) and HFD (high-fat diet and sedentary group) groups (all *P* values < 0.05), while the serum irisin level was greater in the ND and HFDE groups than in the HFD group (both *P* values < 0.05). The fat mass and percentage fat mass were significantly lower in the HFDE group than in the HFD group (both *P* values < 0.01), while higher fat mass and percentage fat mass were observed in the HFD group compared to the ND and NDE groups (both *P* values < 0.01). The visceral fat mass was significantly lower in the HFDE group than in the HFD group (*P* < 0.01); however, a higher visceral fat mass was found in the HFD group compared to those in the ND (*P* < 0.05) and NDE groups (*P* < 0.01) (Fig. 2). *, *p* < 0.05 compared with ND and **, *p* < 0.01 compared with ND. #, *p* < 0.05 compared with NDE and ##, *p* < 0.01 compared with NDE. £, *p* < 0.05 compared with HFD and ££, *p* < 0.01 compared with HFD

**Fig 3:**
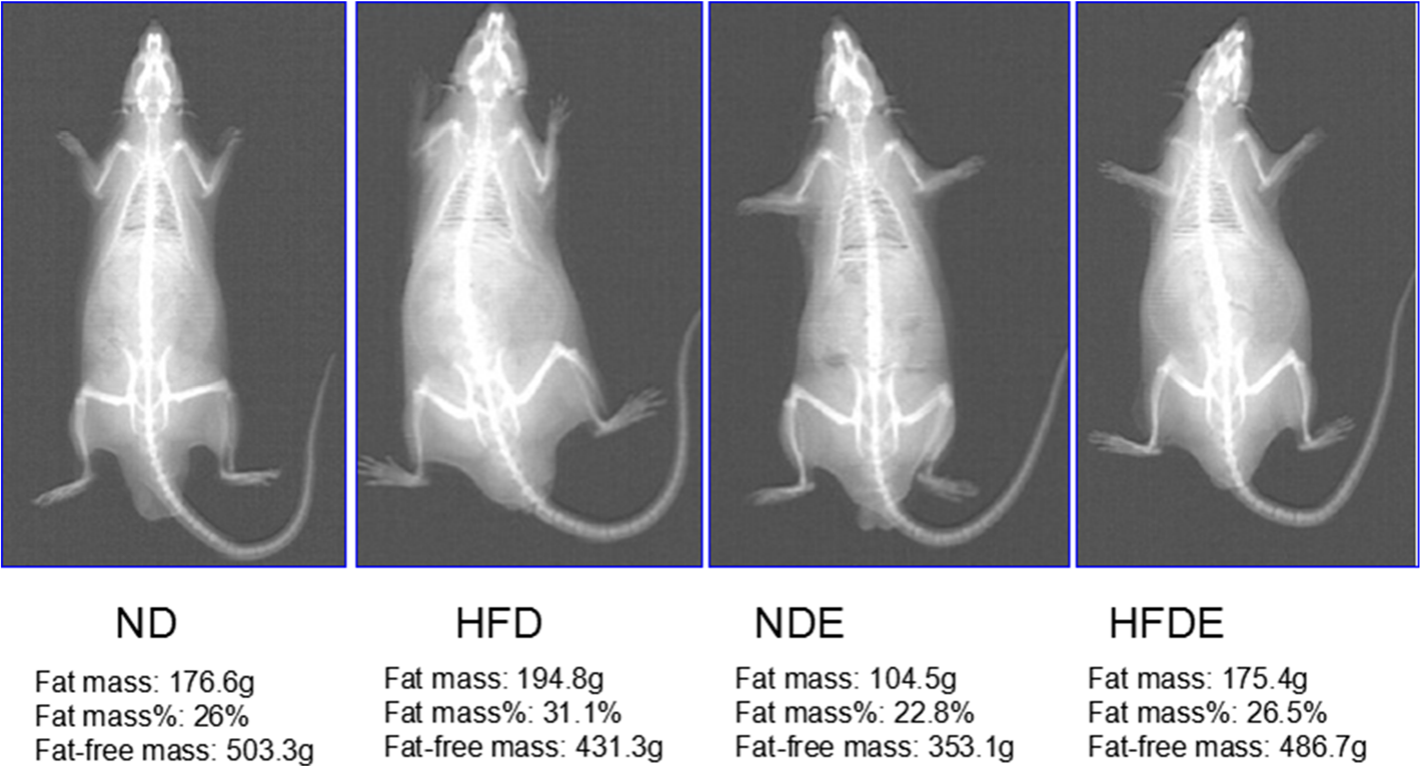
Whole body image of a Wistar rat analyzed with the Hologic in 4 groups at 24 weeks

Dual-energy X-ray absorptiometry (DXA) clearly displayed the fat mass, percentage fat mass and fat-free mass of the rats (Fig. 2). The fat mass and percentage fat mass were significantly lower in the HFDE group than in the HFD group (both *P* values < 0.01), while higher fat mass and percentage fat mass were observed in the HFD group compared to the ND and NDE groups (both *P* values < 0.01). The visceral fat mass was significantly lower in the HFDE group than in the HFD group (*P* < 0.01); however, a higher visceral fat mass was found in the HFD group compared to those in the ND (*P* < 0.05) and NDE groups (*P* < 0.01) (Figs. 2 and 3).

### Association of serum irisin level with obesity-associated parameters

Pearson correlation analysis showed that serum irisin level negatively correlated with TG level (*r* = −0.771, *P* < 0.01), percentage fat mass (*r* = −0.68, *P* < 0.01), fat mass (*r* = −0.576, *P* < 0.01), visceral fat mass (*r* = −0.439, *P* < 0.05) and TC level (*r* = −0.389, *P* < 0.05) in all four groups (Table 1).

**Table 1:** Pearson correlation analysis between serum irisin level and obesity-associated parameters

	The correlation coefficient	*P*
TG(24th)	−0.771	0.00
TC(24th)	−0.389	0.045
Fat mass	−0.576	0.00
Fat mass%	−0.680	0.00
Visceral fat mass	−0.439	0.022

## Discussion

Obesity is primarily a consequence of unhealthy diet and lack of physical activity and the modern best-practice treatment of obesity includes a low-fat diet, increasing physical activity and behavior modification. A recent study found the use of functional foods which have been reported to reduce the overall cardiovascular risk induced by dyslipidemia by acting in synergy with statins [4]. Exercise is an effective approach for prevention and treatment of obesity which could improve multiple indexes of human body. PGC-1α, which was first described in 1998, is reported to strongly correlate with adaptations induced by exercise training [5]. Subsequently, it was found that exercise induced PGC-1α expression in skeletal muscle, which promoted FNDC5 expression, following which FNDC5 was spliced *in vivo* to a new form, irisin [2, 6]. It has been reported that irisin induces browning of subcutaneous white adipocytes, and enhances energy expenditure and fat oxidation. Irisin is therefore identified as a novel target for prevention and treatment of obesity [7].

Our findings showed the body weight, abdominal circumference, Lee’s index, TG and TC levels were higher in high-fat-fed rats, suggesting that rats fed a high-fat diet had characteristics of obese rat models. After 8 weeks of swimming exercise (at 24 weeks), Lee’s index, TG and TC levels were all lower in HFDE group compared with HFD group and the levels of Lee’s index, TG, LDL and insulin reduced in the HFDE group before and after exercise. These findings indicated 8 weeks of swimming exercise reduced Lee’s index and TG level in obese rats fed a high-fat diet, which is consistant with previous studies [3]. The reduction of Lee’s index and TG level is mainly attributable to exercise-induced energy consumption. The decomposition and metabolism of fat produces energy, which is released in the form of heat, thereby resulting in a weight-lowering action.

Recently, it has been established that irisin plays a significant role in energy metabolism and glucose tolerance and, further, that irisin can change the browning of adipose tissue in exercise subjects [8, 9]. However, the effect of exercise training on irisin level remains controversial. Boström et al. [2] reported that irisin expression was dependent on endurance exercise, and irisin level was elevated after 3 weeks of free-wheel running in mice, while 10 weeks of endurance training caused a 2-fold increase in irisin level in healthy adults. In addition, a several-fold increase of irisin level was detected in male athletes compared to middle-aged obese women, and transient exercise increased blood irisin level, while post-surgical body weight reduction decreased muscle FNDC5 expression and blood irisin level [10]. It is notable that high-fat diet reduced serum irisin level while swimming exercise increased irisin level.

Irisin is known as exercise induced myokine, many studies carried out inconsistent results [10–14]. In our study the serum irisin level was highest in the NDE group and lowest in the HFD group, and a higher serum irisin level was detected in the HFDE group relative to the HFD group, suggesting that a high-fat diet reduced serum irisin level while swimming exercise increased irisin level. In agreement with our findings, a recent study observed a significant increase of *FNDC5* mRNA expression in skeletal muscle following a 12-week course of combined strength and endurance training, with significantly higher elevation in a group with pre-diabetes and overweight compared to normoglycemic and normal weight male subjects [13]. Several other studies on human beings have indicated that the effect of exercise on irisin level was associated with exercise intensity and type. High-intensity and resistance exercise has greater improvement on irisin level than low-intensity exercise [11], while resistance exercise has a more marked effect on elevation of circulating irisin levels comapred with high-intensity interval exercise, continuous moderate-intensity exercise groups [12]. But there are also studies that have different results. Interestingly, the irisin levels were increased in acute training but remained unchanged in 8 weeks of training of healthy adults [10]. Recently, Loffler et al, [15] reported that increase irisin levels after acute strenuous exercise and 30-min bout of intensive exercise in children and young adults, whereas longer or chronic increases in physical activity did not affect irisin levels. Timmons and colleagues reported no significant difference in muscle *FNDC5* mRNA expression between either sedentary compared to endurance exercise-trained younger subjects or sedentary versus resistance-trained subjects [14]. There were differences compared to our experiments: 1) type of exercise (most other animal studies applied treadmill exercise, whereas we applied swimming training). 2) subjects (other studies recruited mice or human beings, whereas we recruited male Wistar rats). Therefore, more studies are needed to investigate the variety type of exercise on circulating irisin in human beings and different kinds animals.

Previous studies are rather controversial and there is no general agreement about circulating irisin levels and their correlation with body mass and obesity-associated parameters [16, 17] and the beneficial effect of irisin on the treatment of obesity has been challenged by recent reports in obese animals and humans. Moreno-Navarrete *et al*. (2013) found that circulating irisin correlated negatively with body mass index (BMI), waist-hip ratio and fat mass in men, while Yan and colleagues [18] found that waist circumference was negatively associated with serum irisin. Other reports have reported conflicting data indicating a lack of correlation between circulating irisin concentration and adiposity parameters.

To our knowledge, this is the first study used DXA to measure fat mass, percentage fat mass and fat-free mass in the rats and the measurement of fat parameters is bemore accurate than bioelectrical impedance [19], which was consistent with the findings from the present study (as shown in Fig. 1). The fat mass and percentage fat mass were lower in the HFDE group than in the HFD group, while the fat mass and percentage fat mass were significantly higher in the HFD group than in the ND and NDE groups. In addition, the visceral fat mass was lower in the HFDE group than in the HFD group, and a higher visceral fat mass was detected in the HFD group relative to the ND group and NDE group. Following swimming exercise, Lee’s index, TG, LDL and insulin levels were lower in the HFDE group compared to those before swimming. We observed different results in the Wistar rats in the NDE and HFDE groups following 8-week exercise training of the same type, duration and intensity, under identical dietary conditions. Swimming training could increase serum irisin level in both groups, but a larger rise in serum irisin level was observed in the NDE group than in the HFDE group. In addition, swimming exercise reduced TG level, fat mass and visceral fat mass, and larger reductions in these parameters were found in the HFDE group relative to the HFD group. Serum irisin level correlated negatively with TG level, percentage fat mass, fat mass, visceral fat mass and TC level. These findings demonstrate that irisin may play a specific role in the improvement of fat metabolism with exercise, indicating that irisin accelerates energy consumption of fat tissues.

The results of this study demonstrate that a high-fat diet increases body fat mass and reduces serum irisin level in Wistar rats, and swimming exercise increases serum irisin level in Wistar rats and decreases body fat mass in rats fed a high-fat diet, indicating that the reduction of body fat mass resulting from swimming exercise may be attributable to elevation of serum irisin. We therefore hypothesized that stimulating the secretion of irisin by muscle cells, or artificial synthesis and application of irisin may be promising for the prevention and treatment of metabolic diseases including obesity, which deserves further investigation.

The current study has two limitations. First, we only obtained data pertaining to serum irisin level and fat parameters measured with DXA at the end of the 8-week swimming exercise trial (at 24 weeks of diet feeding), and there is lack of paired comparisons prior to, during and after swimming exercise due to excessive worry about infection of rat tails during swimming. Second, we have no data on FNDC-5and UCP-1 levels in skeletal muscle or fat mass.

## Conclusions

In summary, swimming exercise increases serum irisin levels in Wistar rats and decreases body fat mass in high-fat-diet fed rats, which may be attributable to elevated irisin level caused by swimming exercise.

## Methods

### Animals and grouping

Forty 8-week-old healthy male rats of the Wistar strain were purchased from the Comparative Medicine Center of Yangzhou University (Yangzhou, China). After being raised in the Laboratory Animal Center of the Key Laboratory on Technology for Parasitic Disease Prevention and Control, Ministry of Health, China (Wuxi, China) for 1 week at 24 °C in 50 % humidity, the rats were randomly assigned to one of 4 groups: ND group, NDE group, HFD group, and HFDE group, consisting of 10 animals in each group. All rats were housed in a facility at 18 to 26 °C with 50 % humidity, fed at morning and evening in divided cages, and given free access to water, which was exchanged every other day. During the study period, each rat’s body weight, body size and abdominal circumference were measured every 4 weeks, while blood pressure, heart rate, serum lipid level, blood glucose level and serum insulin level were determined every 8 weeks,

### Animal feeds

Normal rodent feed and high-fat and high-salt feed were purchased from Shanghai Laboratory Animal Co., Ltd. (SLAC; Shanghai, China). The energy composition of normal feed comprises 10 % fat, 22 % protein, 68 % carbohydrate and 0.5 % salt, while the energy composition of the high-fat and high-salt feed consists of 49 % fat, 21 % protein, 30 % carbohydrate and 2 % salt.

### Measurement of obesity-associated parameters

Each rat’s body weight, body size and abdominal circumference were measured every 8 weeks. AC was assessed on the largest zone of the rat abdomen using a plastic non extensible measuring tape, and body size is defined as the length from nose to anus. Lee’s obesity index was estimated using the following formula: Lee’s index = cube root of body weight (g)/body size (cm). After rats were sacrificed, the fat tissues were sampled from the mesentery, bilateral paratesticular region and bilateral perirenal region and weighed, and the gross weight was defined as visceral fat mass. After a 12-h fast, blood samples were collected from rat tails, and FBG, and serum levels of TC, TG, high-density lipoprotein (HDL), and LDL were measured every 8 weeks on a Hitachi 7600 automatic biochemical analyzer (Hitachi; Tokyo, Japan) following the manufacturer’s instructions.

### Swimming training

Swimming exercise was performed without a load in a barrel filled with water at 33–35 °C to a depth of 40–50 cm, which allowed free swimming [20]. The duration of the first swimming exercise was limited to 15 min and increased by 5 min daily up to 30 min. Rats in the NDE and HFDE groups swam for 30 min a day, 5 days a week for 8 successive weeks (16 to 24 weeks), while animals in the ND and HFD groups were sedentary in cages.

### Determination of serum insulin concentration

Serum insulin concentration was determined using a chemiluminescence immunoassay (CLIA) kit on a Unicel Dxi800 access immunoassay system (Beckman Coulter, Inc.; Brea, CA, USA). This assay showed -0.26 % cross-reactivity to proinsulin, and intra-assay and inter-assay coefficients of variation (CVs) were 4.2 and 5.5 %, respectively.

### Measurement of serum irisin concentration

Quantitative measurement of irisin in rat serum samples was performed using a commercial enzyme-linked immunosorbent assay (ELISA) kit directed against amino acids 42–112 of the FNDC5 protein (Phoenix Pharmaceuticals, Inc.; Burlingame, CA, USA) according to the manufacturer’s instructions. Absorbance from each sample was measured in duplicate using a VersaMax microplate reader (Associates of Cape Cod, Inc.; East Falmouth, MA, USA) at a wavelength of 450 nm.

### Dual-energy X-ray absorptiometry (DXA)

All rats were administered 1.5 % pentobarbitone at a dose of 40 mg/kg injected intramuscularly for induction of anesthesia. Rats were placed in a prone position and scanned by DXA using the Hologic ultra-high resolution rat whole body composition software. The duration of each scan was 3 to 5 min, and all scans were processed with ultra-high resolution analysis software. Body fat mass, percentage of fat mass and fat-free mass were recorded [21].

### Ethical consideration

All animal studies were conducted in accordance with the recommendations in the Guidelines for the Care and Use of Laboratory Animals of the Ministry of Science and Technology of the People's Republic of China ([2006]398). This study was approved by the Ethics Review Committee of Jiangsu Lake Taihu Cadres Sanatorium (Permission number: SGLERC-2011008) and Jiangsu Provincial People’s Hospital Group (Permission number: SRY20100820).

### Statistical analysis

All measurement data are expressed as mean ± standard deviation (SD), and all statistical analyses were performed using the statistical software SPSS version 17.0 (SPSS Inc.; Chicago, IL, USA). Differences between the means were tested for statistical significance with one-way analysis of variance (ANOVA) and Student’s *t*-test; intra-group comparisons before and after swimming exercise were carried out using the paired *t*-test. The association of serum irisin level with obesity-related parameters was evaluated using the Pearson correlation analysis. A *P* value < 0.05 was considered statistically significant.

## Abbreviations

AC: abdominal circumference
BMI: body mass index
CLIA: chemiluminescence immunoassay
CV: coefficients of variation
DXA: dual-energy x-ray absorptiometry
ELISA: enzyme-linked immunosorbent assay
FBG: fasting blood glucose
HDL: high-density lipoprotein
HFD: high-fat diet and sedentary
HFDE: high-fat diet and exercise
LDL: low-density lipoprotein
ND: normal diet and sedentary
NDE: normal diet and exercise
PGC-1α: proliferator-activated receptor gamma coactivator 1 alpha
TC: total cholesterol
TG: triglyceride
